# Cohort differences in the levels and trajectories of frailty among older people in England

**DOI:** 10.1136/jech-2014-204655

**Published:** 2015-02-02

**Authors:** Alan Marshall, James Nazroo, Gindo Tampubolon, Bram Vanhoutte

**Affiliations:** Cathie Marsh Institute for Social Research, University of Manchester, Manchester, UK

**Keywords:** AGEING, INEQUALITIES, LONGITUDINAL STUDIES, Cohort studies

## Abstract

**Background:**

The level of frailty in the older population across age cohorts and how this changes is a factor in determining future care costs and may also influence the extent of socioeconomic and gender inequalities in frailty.

**Methods:**

We model cohort-specific trajectories in frailty among the community dwelling population older than 50 years, using five waves (2002–2010) of the English Longitudinal Study of Ageing. We stratify our analysis by wealth and gender and use a frailty index, based on accumulation of ‘deficits’.

**Results:**

For males and females between the ages of 50 and 70 in 2002, frailty trajectories for adjacent age cohorts converge. However, levels of frailty are higher in recent compared with earlier cohorts at the older ages (for cohorts aged over 70 in 2002). These cohort differences are largest in the poorest wealth group, while for the most affluent, frailty trajectories overlap across all adjacent cohorts suggesting no change across cohorts.

**Conclusions:**

A key driver of the cohort differences in frailty that we observe is likely to be increased survival of frail individuals. Importantly, this paper illustrates that the social conditions experienced across the wealth distribution impacts on the rate of deficit accumulation in older populations. Our results on trajectories of frailty between 2002 and 2010 are pessimistic and, in the context of rising life expectancies, suggest that poorer older people in particular spend additional years of life in a frail state.

## Introduction

A fundamental question within ageing research is whether the extension of life expectancy has been accompanied by longer or shorter periods of healthy life expectancy. Three theories have been proposed.^1–3^ The compression of morbidity theory argues that the onset of morbidity is delayed so that as life expectancy increases people spend fewer years of their life in poor health.^1^ It is argued that the factors (such as improved nutrition, medical advances and improvements in care, and less physically arduous working conditions) that drive the trend of increasing longevity *also* delay the age at onset of disabling conditions and slow the progression of such conditions. As a result the time spent in poor health is compressed to the final years of life.^1^

In contrast, the expansion of morbidity theory gives a pessimistic outlook under which the age of poor health onset remains roughly constant (or lags improvements in longevity) so that gains in life expectancy are composed mainly of increasing years spent with chronic health problems.^2^ The mechanism underlying this theory is that improvements in modern medicine extend the lives of those with chronic health problems and disabilities to a greater degree than they slow the onset and progression of such conditions.^2^ Additionally, it is argued that where there are rises in unhealthy lifestyle choices and associated conditions, such as obesity, in younger compared with older cohorts, these may have serious implications for age at onset of chronic health problems, such as diabetes, providing an additional contribution to the proposed expansion of morbidity.^4^

Clearly, there is scope for aspects of both theories to operate and the receding horizon model falls between the expansion and compression of morbidity models and proposes that the onset and progression of morbidity is postponed to exactly the same extent as death so that the number of years spent in poor health remains unchanged.^3^

The findings of research into this issue vary according to the health outcome, the type of data used (cross-sectional or longitudinal), social class and across countries, perhaps reflecting different social, cultural and political contexts.^4–11^ Furthermore, there is considerable uncertainty as to how health trajectories at older ages might be affected by the changing prevalence of risk factors such as obesity, sedentary lifestyles, smoking and alcohol consumption.^4^ A weakness of existing studies, particularly in the UK, is that they have tended to draw on repeated cross-sectional data,^12^ ^13^ which is problematic in terms of the separation of age and cohort effects.

In this paper we model trajectories of frailty among older, community-dwelling people in England over the last decade (2002–2010) focusing on inequalities according to gender and wealth. A central aspect is the identification of cohort differences: do people have a better (or worse) frailty score relative to their counterparts in different cohorts but at an equivalent age. Cohort improvements in health over time have the potential to reduce the costs associated with a more elderly population,^14^ ^15^ yet if these are concentrated among the affluent they will serve to exacerbate the extent of health inequality. Our focus on frailty, a multidimensional measure of health, offers a valuable contribution to the existing research on trends in health that has tended to examine indicators of single aspects of health.

Frailty has emerged as a key aspect in research on population ageing and geriatric clinical practice. While specific definitions and measures of frailty are contested, there is general agreement that frailty is a non-specific state reflecting age-related declines in multiple systems, which lead to a range of adverse outcomes such as falls, fractures, hospitalisation, institutionalisation and mortality.^16^ Frailty is a useful outcome with which to consider the challenges of caring for a growing elderly population; it provides an indication of an individual’s capacity for independent living and the risk of suffering an adverse event, such as a fall, that might precipitate a need for greater levels of care in the future. A number of measures of frailty have been developed, including a *frailty index* which is based on the number of ‘deficits’ held by an individual and which has been validated as a predictor of mortality and institutionalisation in the literature.^16–19^

In this paper we explore whether levels of dependency are changing across cohorts by examining differences in trajectories of frailty including stratification by socioeconomic position and gender. We use a frailty index derived using data from the English Longitudinal Study of Ageing (ELSA) and study the period 2002–2010.

## Methods

### Background

We model frailty trajectories among older people in the community (age 50+). Each frailty trajectory comprises an estimate of the mean level of frailty in 2002 and the subsequent change in frailty over an 8-year period up to 2010. We derive distinct frailty trajectories for seven cohorts based on an individual's age in 2002 (50–54, 55–59, 60–64, 65–69, 70–74, 75–79 and 80+). The cohort-specific frailty trajectories overlap over the study period, enabling comparison of the level and rate of change of frailty across cohorts at equivalent ages (but different points in time). We consider whether the cohort-specific trajectories converge, suggesting that levels of frailty are unchanged across cohorts, or diverge, suggesting improvement/deterioration in levels of frailty across cohorts. Finally, we consider whether any cohort differences in frailty vary according to gender and across wealth distribution.

### Data

We use data from five waves of the ELSA collected between 2002 and 2010. ELSA is a representative sample of the population aged 50 and over, living in private households in England and is a rich data source containing information on sociodemographic characteristics, health, social participation and biomarkers.^20^

The ELSA sample size at baseline in 2002 comprises 11 391 individuals (aged 50+) representing a response rate of 67%. Of this sample, 11 220 participants (98%) have sufficient non-missing values across deficit variables (30 or more of 60) for a frailty index to be calculated. Encouragingly, over 95% of the sample responded to at least 58 of the 62 deficits in each wave of the survey, a level of item response that holds for men and women and across the wealth distribution. As for most longitudinal studies, ELSA suffers from a degree of attrition (see online supplementary information and table S1 for further details) as a result of a range of factors such as mortality, non-response, migration overseas, illness and institutionalisation, so that by wave 5 the sample size is reduced to 6242. ELSA has an average of 3.6 observations per individual (of a possible total of five).

The rich detail of the ELSA data set enables production of a frailty index including 60 items covering a range of domains (activities of daily living, cognitive function, falls and fractures, joint replacement, vision, hearing, chronic diseases, cardiovascular diseases and depression). Following guidance in the literature we only include individuals in our study if they have non-missing values for at least 30 of the 60 frailty components.^21^ A full list of the deficits and their coding is provided in the online supplementary material.

The key explanatory variables in this paper are an individual’s age in the first wave of ELSA (2002), according to quinary age groups (cohort identifier), gender and wealth. This wealth variable includes the net financial and physical wealth and the net housing wealth for each household. We utilise a measure of household rather than individual wealth because in the vast majority of cases older couples deal with finances jointly. Pension wealth is known to be particularly age dependent, declining throughout an individual’s retirement^22^ and so we exclude it from our analysis. We calculate wealth tertiles for each cohort separately at wave 1 in order to accommodate cohort-specific differences in financial circumstances. The sample characteristics at waves 1 and 5 are given in table 1; the age and sex structure of the ELSA sample at wave 1 (2002) is in line with the household population in the 2001 census. The mean level and distribution of frailty is comparable with that observed in other similar studies.^18^ ^21^

**Table 1:** Sample characteristics at the first and fifth wave of English Longitudinal Study of Ageing

	Wave 1 (2002) Cohort (based on age in 2002)															
	All (N=11 220)		50–54 (1966)		55–59 (2164)		60–64 (1666)		65–69 (1687)		70–74 (1452)		75–79 (1078)		80+ (1207)	
	M/%	SD	M/%	SD	M/%	SD	M/%	SD	M/%	SD	M/%	SD	M/%	SD	M/%	SD
Frailty Index	0.16	0.12	0.12	0.11	0.14	0.11	0.15	0.11	0.16	0.11	0.18	0.11	0.20	0.12	0.24	0.12
Age	65	10	52	1	57	1	62	1	67	1	72	1	77	1	84	4
Female	54%	50	55%	50	53%	50	52%	50	53%	0.50	54%	0.50	54%	50	62%	49
Poorest tertile	33%	47	33%	47	33%	47	33%	47	33%	47	33%	47	33%	47	33%	47
Middle wealth tertile	33%	47	33%	47	33%	47	33%	47	33%	47	33%	47	33%	47	33%	47
Richest wealth tertile	33%	47	33%	47	33%	47	33%	47	33%	47	33%	47	33%	47	33%	47

	Wave 5 (2010) Cohort (based on age in 2002)															
	All (N=6237)		50–54 (1298)		55–59 (1409)		60–64 (1307)		65–69 (1030)		70–74 (727)		75–79 (451)		80+ (284)	
	M/%	SD	M/%	SD	M/%	SD	M/%	SD	M/%	SD	M/%	SD	M/%	SD	M/%	SD
Frailty Index	0.18	0.13	0.13	0.11	0.15	0.11	0.17	0.12	0.19	0.12	0.23	0.13	0.27	0.14	0.34	0.16
Age	71	9	61	1	65	2	70	2	75	1	80	1	85	2	91	2
Female	56%	49	56%	50	54%	50	55%	50	56%	50	57%	50	59%	49	68%	46
Poorest tertile	28%	45	30%	46	28%	45	28%	45	27%	45	28%	45	25%	44	24%	43
Middle wealth tertile	34%	47	32%	47	36%	48	34%	47	35%	48	35%	48	34%	47	32%	47
Richest wealth tertile	38%	48	37%	48	36%	48	38%	49	37%	48	37%	48	41%	49	44%	50

### Model

We fit a series of multilevel growth curve models that predict the level of frailty in wave 1 (2002) and the subsequent change in frailty between waves 1 and 5 (2002–2010) dependent on cohort at wave 1. Our multilevel model consists of repeated observations nested within individuals, offering one way of dealing with the non-independence of an individual’s level of frailty over time. A key advantage of using a multilevel approach is that the technique is capable of handling unequal time spaces, missing data, and the inclusion of time varying and between-subject covariates that are either continuous or discrete measures.^23^ We use a similar methodology to that in a study of depression in later life^24^ to investigate whether cohorts share the same or different frailty trajectories. We extend this methodology to consider whether cohort-specific trajectories differ by gender and wealth.

The models fitted are specified below (equations 1–5) where FI_ti_ indicates the frailty index of individual i at time t for i=1…11 220 and t=1…t_i_; t_i_ is the number of repeated measures available for individual i and ranges from 1 to 5 depending on the number of survey waves that an individual participated in (see online supplementary material for more details on missingness). In the level 1 repeated measures model (equation 1), each individual's frailty trajectory is modelled as a function of time, where time_ti_ denotes the timing of occasion t for individual i and is based on a centred version of survey wave (time=−2, −1, 0, 1, 2; see elsewhere for details on variable centring^25^). We include a quadratic term, 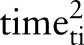, to allow for the development of non-linear frailty trajectories. Thus, in equation 1, the intercept β_0i_ gives the mean frailty index of person i in wave 3 while β_1i_ and β_2i_ give the linear and quadratic growth of frailty over time for individual i. In equation 1, e_it_ is the random within-person error for person i at time t, which is assumed to be normally distributed. The level 2 model (growth trajectories across individuals) allows for a distinct trajectory for each age cohort (equations 2 and 3). Here, cohort_i_ indicates the cohort membership of individual i and is treated as a (centred) continuous variable taking the values −3, −2, −1, 0, 1, 2 and 3. Operationalisation of cohort using dummy variables gives similar results and identical conclusions. We experimented with inclusion of a cohort squared term but this was not statistically significant so we did not include it in the models reported here. In equation 2, γ_00_ is the mean frailty index at wave 3 of cohort 0 (65–69 years old in 2002).

Level 1 model (growth trajectory within individuals)1
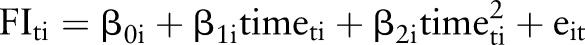


Level 2 model (growth trajectory across individuals)

For the intercept:2
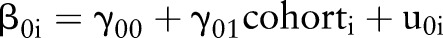


For the slope:3
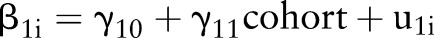


We develop the model above to allow frailty growth trajectories for cohorts to vary according to gender and wealth tertiles (both treated as time invariant). This involves the same level 1 model as specified in equation 1 but equations 2 and 3 are adapted to include an interaction between cohort and either wealth or gender (see example below for gender differences in frailty trajectories).4
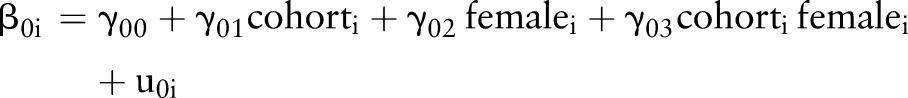
5
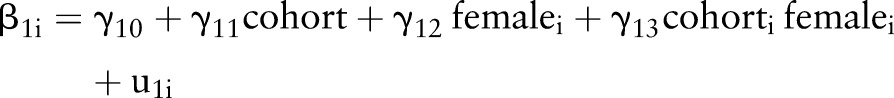


All analyses are carried out in Stata/SE12.1. We include wave 1 cross-sectional weights to ensure that the sample is representative of the community-dwelling population aged over 50 in 2002. We also conducted sensitivity analysis to assess the impact of attrition on our findings by fitting the same models using only those who participated in all five waves of ELSA, which enabled us to use the provided survey weights that adjust for attrition between waves 1 and 5. This sensitivity analysis (see online supplementary material) did not affect any of our conclusions and is referred to in the discussion.

## Results

**Figure 1:**
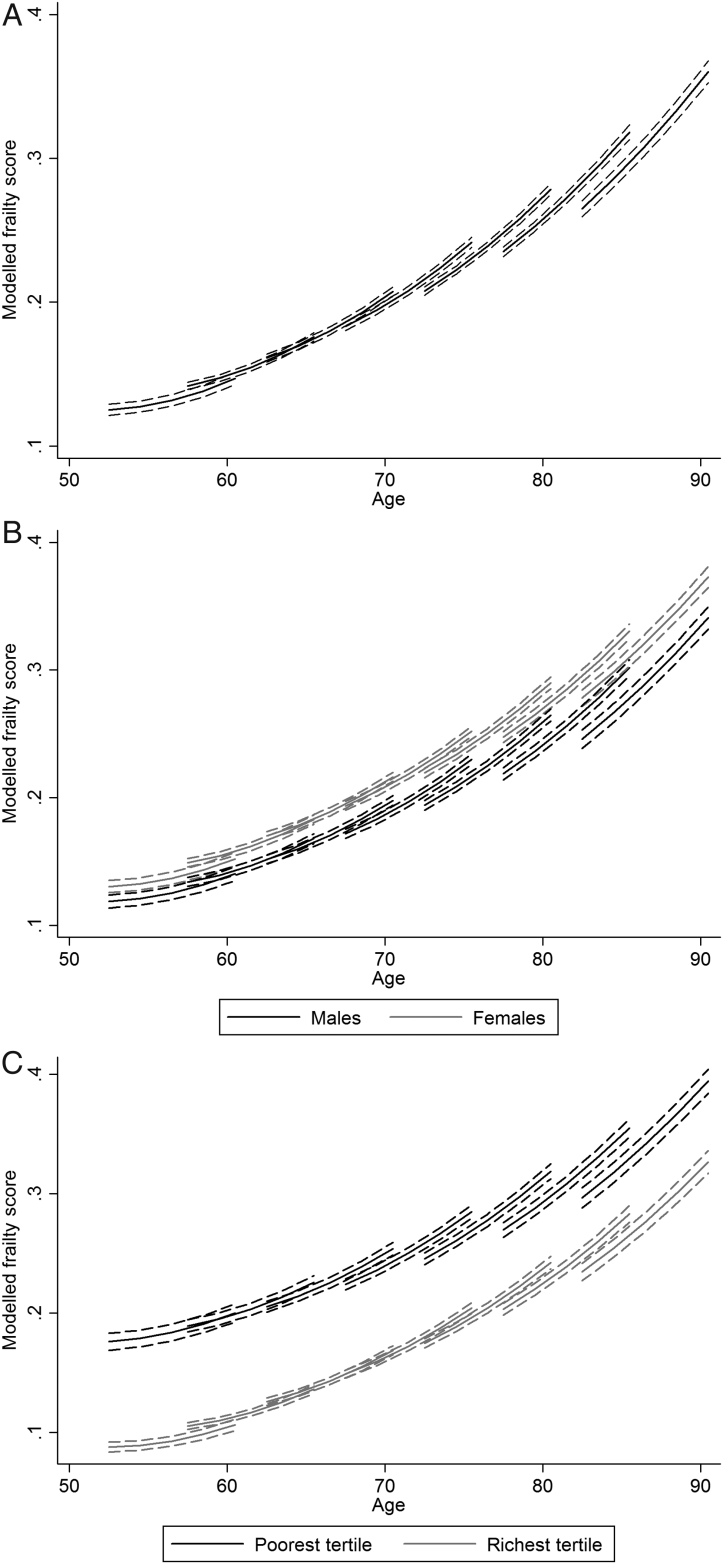
Frailty trajectories: (A) all people (aged 50+); (B) by gender and (C) by wealth.

Figure 1A–C displays vector graphs of the model frailty trajectories with 95% CIs represented by dotted lines. The estimated coefficients that specify the trajectories are given in the online supplementary material. In this paper, each line represents a frailty trajectory for a particular cohort, which starts at the age midpoint for that cohort in 2002 and then tracks the change in frailty over the next 8 years until 2010. In figure 1A, we observe little evidence of difference in levels of frailty across the recent cohorts (from age 50 to 70 when first observed in 2002). However, there does appear to be a difference in frailty across the earlier cohorts (starting from the age of 70 when first observed in 2002) with higher frailty in recent cohorts compared with earlier cohorts. The same slope of frailty and the patterns of cohort differences in frailty hold for men and women (see figure 1B) suggesting that the differential of higher frailty in women compared with men did not change between 2002 and 2010.

The wealth differences in levels of frailty shown in figure 1C are stark; the average trajectory of frailty for an individual in the richest tertile at age 80+ is comparable with that for an individual at age 70–74 in the poorest tertile. Figure 1C suggests that inequalities in levels of frailty widened between 2002 and 2010, at particular overlapping ages recent cohorts appear to have higher levels of frailty compared with earlier cohorts among the poorest tertile (take, eg, levels of frailty between the ages of 75 and 80 for the cohort aged 70–74 in 2002 and the cohort aged 75–79 in 2002), while for the richest there is little difference in frailty across cohorts. A key driver of this wealth-specific cohort difference is the slower estimated growth rate of frailty for those in the richest tertile compared with the poorest tertile (the interaction coefficient: richest tertile×growth rate=−0.001; p=0.03).

## Discussion

The analysis presented reveals a pessimistic set of findings concerning trends in frailty between 2002 and 2010. There is no evidence for improvement in levels of frailty across cohorts, with a strong suggestion of higher levels of frailty among recent cohorts compared with earlier cohorts (for those cohorts aged over 70 in 2002), particularly for the poorest individuals. This carries the negative implication that the continuing gains in life expectancy are composed of additional years spent in frailty with the potential for greater associated healthcare costs. Inequalities in frailty across the wealth distribution are stark and they appear to widen over the period 2002–2010; the increase in frailty in recent cohorts compared with earlier cohorts is most prominent among the poorest individuals with little evidence of such cohort differences among the most affluent. Levels of frailty are higher for women than for men and the analysis of cohort and gender-specific trajectories suggests that this difference has remained unchanged over the study period.

The lack of evidence for improvement in levels of frailty across most cohorts is in line with other UK-based research (cross-sectional and longitudinal) that has found limited evidence for improvements in various health outcomes from 1990s to the present.^12^ ^13^ ^26–28^ A US-based study that examined cohort-specific trajectories of frailty also reported higher levels of frailty in recent cohorts compared with earlier cohorts^29^ and similar findings are observed in the USA for cohort-specific trajectories of self-reported illness.^30^ ^31^ Our finding of different slopes of frailty across cohorts is in line with a US-based study of depression in later life.^24^ However, this study reported cohort differences in levels of depression in the opposite direction to our findings for frailty with higher levels of depression in earlier cohorts compared with recent cohorts. This illustrates the potential for variability in cohort differences across different domains of health.

The higher frailty we observe in recent cohorts compared with earlier cohorts (for cohorts aged over 70 in 2002) may reflect improvements in medical and care services across the life course that increase the survival of frail individuals with the effect most prominent at the oldest ages where risks of frailty are highest. Additionally, these cohort differences in frailty might also reflect real deterioration in the condition of older people over time separate to any improvement in life expectancy. One mechanism might be increases in the prevalence of unhealthy lifestyle choices, which are concentrated in recent cohorts compared with earlier cohorts. If such unhealthy lifestyle choices have a cumulative effect over the life course and are socially patterned then they have the potential to explain the nature of the cohort differences that we observe, which are strongest at the oldest ages and for the poorest individuals. Analysis of ELSA data between 2004 and 2008^32^ reveals mixed evidence for this theory. First, while some unhealthy lifestyle choices are most prevalent for the poorest individuals (such as inactivity, fruit and vegetable consumption and smoking), for others the social patterning is less clear (such as alcohol consumption and obesity). Second, not all unhealthy lifestyle choices are increasing in the age cohorts covered here. Comparison of the cohort-specific extent of smoking and daily alcohol consumption within ELSA reveals *declines* in prevalence between 2004 and 2008, although there was an increase in the prevalence of sedentary lifestyles and waist circumference over this period. The review above points to the need for further analysis on cohort-specific trends in specific unhealthy lifestyle choices (including prior to the period of this study) in order to fully understand their impact on the cohort differences in later life frailty. We also need to consider the drivers of such changes in lifestyle.

Social conditions appear to influence the rate of deficit accumulation in older populations, as has been noted in the USA.^29^ We observe faster cohort-specific growth in frailty among poorer compared with richer individuals, widening the strong inequalities observed in frailty at the start of the study in 2002. This finding is supported by recent evidence on socioeconomic trends in disability-free life expectancy at the older ages in England; for example, at age 65, the years spent with a disability increased between 2002–2005 and 2006–2009 in the most deprived neighbourhoods but remained constant in the least deprived neighbourhoods.^33^ Income inequality in England increased steeply during the 1980s and has been sustained throughout the 1990s and 2000s at historically high levels.^34^ A body of literature claims that the growth in health inequality reflects trends in wealth inequality.^35^ The accumulation of stress and disadvantage relating to social position across the lifecourse^36^ combined with the growth in economic inequality in England from the 1930s offers another explanation for the cohort differences observed here for the poorest older people. As has been previously predicted elsewhere,^34^ our analysis reveals higher levels of frailty at the oldest ages for recent cohorts compared with earlier cohorts, perhaps because they have lived through a time of greater inequality with greater psychological penalties associated with low social position compared with later cohorts. While our results show that the extent of the inequality in frailty across wealth tertiles is smallest at the oldest ages, it is probable that selective mortality effects offer one explanation for this observation.^37^

In this paper, as in other studies,^38^ we find higher levels of frailty for women compared with men. The growth of frailty for cohorts is identical for men and women mirroring the gender-specific dynamics of frailty observed in the USA.^29^ The absence of differential frailty cohort effects between men and women suggests that gender inequalities in frailty have not diminished between 2002 and 2010. This stability in gender frailty inequality is in line with that observed for physical health and self-rated health (but not depression).^30^

This paper is subject to some limitations. First, although accelerated cohort studies are regarded as the gold standard in terms of analysing changes in health and functioning over time and separating effects of age and cohort, they are subject to an important limitation regarding the possibility that cohort effects are actually attributable to period effects. Models that attempt to separate age, period and cohort effects, including recent developments, have been subjected to strong criticism,^39^ with some arguing that it is impossible to separate all three influences.^40^ We cannot exclude the possibility that a period effect has some influence on the cohort differences we observe. However, such a possibility is somewhat reduced given that the period we study is relatively short (2002–2010).

Related to this, it is possible that changes in levels of institutionalisation at the older ages might influence our results. In England there has been a slight shift towards a greater proportion of older people remaining within the community rather than living in institutions between 2002 and 2010. Thus, at a particular age, it is likely that an earlier cohort in wave 1 (2002) had a slightly greater proportion of frail individuals within care homes compared with a recent cohort in wave 5 (2010), where institutional living is slightly less common. However, the level of institutional living at older ages and the change over this period is relatively small; the census estimates reveal that 4.6% of the population aged over 65 were in institutions in 2001 compared with 3.7% in 2011. While levels of institutional living are higher at the oldest ages the levels and change in the proportions living in institutions are modest; 12.6% of the population aged over 80 were in institutions in 2001 compared with 10% in 2011. Thus, the changes in prevalence of institutional living in later life are unlikely to be a key driver of the cohort differences in frailty that we observe.

A further limitation of our study relates to attrition and the tendency for those who drop out of the study to have different characteristics to those who participate fully. Analysis of attrition in ELSA illustrates that those who drop out are most likely to be frail, older (members of an earlier birth cohort), male and poor (see online supplementary material). It is logical that without such attrition we would observe steeper frailty trajectories, from the same starting point (weights for non-response in wave 1 are included in the analysis), particularly so for the poor, earlier birth cohorts and males. Consequently, the impact of attrition on our frailty trajectories is likely to have minimised our main findings; we might expect that accounting for attrition would strengthen the deterioration we observe in frailty across cohorts, making the possibility of improvement in frailty within recent cohorts compared with earlier cohorts even more unlikely. Similarly, the wealth differences in frailty across cohorts are likely to be exacerbated if attrition was accounted for. In separate analysis that uses only those who participated in all five waves of ELSA and that employs longitudinal weights to adjust for attrition between waves 1 and 5, we find comparable cohort differences to those reported in this paper (see online supplementary material).

Health inequalities are considered undesirable for many reasons including fairness, the related adverse impacts on wider society, the avoidable nature of health inequality and cost-effectiveness.^41^ This paper shows that the stark inequalities in frailty in later life may well have widened between 2002 and 2010, and certainly have not reduced. This trend is a concerning one that does not bode well for the future. It points towards a further divergence in health experiences in later life across wealth distribution as life expectancies continue to increase. Effective policy responses to this trend will require a strong political will as well as a clear understanding of the drivers of inequality throughout the life course. Crucially, the challenges of population ageing and health inequalities should not be seen in isolation.

**What is already known on this subject?**

- Frailty at older ages is known to be associated with adverse outcomes such as mortality, dependency and institutionalisation.
- In later life, levels of frailty are higher for women compared with men and there is a strong gradient of decreasing frailty with increasing wealth.
- A recent study in the USA revealed a pessimistic finding of higher levels of frailty in recent cohorts compared with earlier cohorts at an equivalent age.

**What this study adds?**

- This study demonstrates that in England, recent cohorts have higher levels of frailty compared with earlier cohorts of equivalent age from the age of 70 and above.
- The increase in frailty in recent compared with earlier cohorts is most prominent among the poorest individuals with little evidence of any difference in frailty across cohorts among the most affluent.

## Supplementary Material

Web supplement

## References

[1] Fries J Aging, natural death, and the compression of morbidity. N Engl J Med 1980;303:130–5. 7383070 10.1056/NEJM198007173030304

[2] Gruenberg EM The failure of success. Milbank Q 1976;55:3–24. 141009

[3] Manton KG Changing concepts of morbidity and mortality in the elderly population. Milbank Q 1982;60:183–244. 6919770

[4] Crimmins E, Beltran-Sanchez H Mortality and morbidity trends: is there compression of morbidity? J Gerontol B Psychol Sci Soc Sci 2011;66B:75–86. 21135070 10.1093/geronb/gbq088 PMC3001754

[5] Parker M, Thorslund M Health trends in the elderly population: getting better and getting worse. Gerontologist 2007;47:150–8. 17440120 10.1093/geront/47.2.150

[6] Crimmins E, Saito Y Trends in healthy life expectancy in the United States 1970–1990: gender, racial and educational differences. Soc Sci Med 2001;52:1629–41. 11327137 10.1016/s0277-9536(00)00273-2

[7] Freedman V, Crimmins E, Schoeni R, et al Resolving inconsistencies in trends in old-age disability: report from a technical working group. Demography 2004;41:417–41. 15461008 10.1353/dem.2004.0022

[8] Manton K Recent declines in chronic disability in the elderly U.S. population: risk factors and future dynamics. Annu Rev Public Health 2008;29:91–113. 18031222 10.1146/annurev.publhealth.29.020907.090812

[9] Yong T, Saito Y Trends in healthy life expectancy in Japan: 1986–2004. Demogr Res 2009;20:467–94.

[10] Yong T, Saito Y, Chan A Changes in the presence of mobility limitations and mobile life expectancy in Singapore, 1995–2005. J Aging Health 2010;22:120–40. 19934444 10.1177/0898264309351932

[11] Unger R Trends in active life expectancy in Germany between 1984 and 2003—a cohort analysis with different health indicators. J Public Health 2006;14:155–63.

[12] [No authors listed]. Office for National Statistics Report: Health expectancies in the UK, 2002. Health Stat Q 2006;29:59–62. 16523680

[13] Martin L, Schoeni R, Andreski P, et al Trends and inequalities in late-life health and functioning in England. J Epidemiol Community Health 2011;66:874–80. 22147749 10.1136/jech-2011-200251

[14] Spijker J, MacInnes J Population ageing: the time bomb that isn't? BMJ 2012;66:874–80.

[15] Sanderson W, Scherbov S Remeasuring aging. Science 2010;328:p1287–8. 10.1126/science.1193647 20829469

[16] Rockwood K, Mitnitski A, Song X, et al Long-term risks of death and institutionalization of elderly people in relation to deficit accumulation at age 70. J Am Geriatr Soc 2006;54:975–9. 16776795 10.1111/j.1532-5415.2006.00738.x

[17] Rockwood K, Mitnitski A Frailty in relation to the accumulation of deficits. J Gerontol A Biol Sci Med Sci 2007;62A:722–7. 17634318 10.1093/gerona/62.7.722

[18] Ravindrarajah R, Lee D, Pye S, et al The ability of three different models of frailty to predict all cause mortality. Results from the European Male Ageing Study (EMAS). Arch Gerontol Geriatr 2013;57:360–8. 23871598 10.1016/j.archger.2013.06.010

[19] Kulminski A, Yashin K, Arbeev I, et al Cumulative index of health disorders as an indicator of aging associated processes in the elderly: results from analyses of the National Long Term Care survey. Mech Ageing Dev 2007;128:250–8. 17223183 10.1016/j.mad.2006.12.004 PMC1866299

[20] Cheshire H, Hussey D, Phelps A, et al Methodology. In: Dynamics of ageing: evidence from the English longitudinal study of ageing. Banks J, Nazroo J, Steptoe A, eds. London: The Institute for Fiscal Studies, 2012:183–213. ISBN: 978-10-903274-92-7.

[21] Searle S, Mitnitski A, Gahbauer E, et al Standard procedure for creating a frailty index. BMC Geriatr 2008;8:24. 18826625 10.1186/1471-2318-8-24 PMC2573877

[22] Crawford R, Tetlow G The evolution of pension wealth and contribution dynamics. In: The dynamics of ageing: evidence from the English longitudinal study of ageing. Banks J, Nazroo J, Steptoe A, eds. London: Institute for Fiscal Studies, 2012:10–47.

[23] Raudenbush S, Chan W Growth curve analysis in accelerated longitudinal designs. J Res Crime Delinq 1992;22:387–411.

[24] Yang Y Is old age depressing? Growth trajectories and cohort variations in late life depression. J Health Soc Behav 2007;48:16–32. 17476921 10.1177/002214650704800102

[25] Miyazaki Y, Raudenbush S Tests for linkage of multiple cohorts in an accelerated longitudinal design. Psychol Methods 2000;5:44–63. 10937322 10.1037/1082-989x.5.1.44

[26] Jarvis C, Tinker A Trends in old age morbidity and disability in Britain. Ageing Soc 2000;19:603–27.

[27] Rees P, Zuo C, Wohland P, et al The implications of ageing and migration for the future population, health, labour force and households of Northern England. Appl Spat Anal Policy 2013;6:93–122.

[28] Zaninotto P, Nazroo J, Banks J Trends in disability. In: Financial circumstances, health and wellbeing of the older population in England. Banks J, Lessof C, Nazroo J, Rogers N, Stafford M, Steptoe A, eds. London: Institute for Fiscal Studies, 2010:254–74.

[29] Yang Y, Lee L Dynamics and heterogeneity in the process of human frailty and aging: evidence from the U.S. older adult population. J Gerontol B Psychol Sci Soc Sci 2010;65B:246–55. 20007299 10.1093/geronb/gbp102 PMC2981448

[30] Yang Y, Lee L Sex and race disparities in health: cohort variations in life course patterns. Soc Forces 2009;87:2093–124.

[31] Mirowsky J, Ross C Education and self-rated health: cumulative advantage and its rising importance. Res Aging 2008;30:93–122.

[32] de Oliveira C, Shankar A, Kumari M, et al Health risk and health protective biological measures in later life. In: Financial circumstances, health and well-being of the older population in England: the 2008 English longitudinal study of ageing. Banks J, Lessof C, Nazroo J, Rogers N, Stafford M, Steptoe A, eds. London: The Institute for Fiscal Studies, 2010:275–347.

[33] Office for National Statistics. Inequality in disability free life expectancy by area deprivation: England 2002–5 and 2006–9. ONS Stat Bull 2012 http://www.ons.gov.uk/ons/dcp171778_265133.pdf

[34] Shaw M, Davey Smith G, Dorling D Health inequalities and new labour: how the promises compare with real progress. BMJ 2005:330:1016–21. 15860830 10.1136/bmj.330.7498.1016 PMC557156

[35] Shaw M, Dorling D, Gordon D, et al The widening gap: health inequalities and policy in Britain. Bristol: Policy Press, 1999.

[36] Dannefer D Cumulative advantage/disadvantage and the life course: cross-fertilizing age and social science theory. J Gerontol B Psychol Sci Soc Sci 2003;58B:327–37. 10.1093/geronb/58.6.s327 14614120

[37] McMunn A, Nazroo J, Breeze E Inequalities in health at older ages: a longitudinal investigation of onset of illness and survival effects in England. Age Ageing 2009;38:181–7. 19029098 10.1093/ageing/afn236 PMC2724887

[38] Kulminski A, Culminskaya I, Ukraintseva S, et al Sex-specific health deterioration and mortality: the morbidity paradox over age and time. Exp Gerontol 2008;43:1052–7. 18835429 10.1016/j.exger.2008.09.007 PMC2703431

[39] Bell A, Jones K Another ‘futile quest’? A simulation study of Yang and Land's hierarchical age-period-cohort mode. Demogr Res 2014;30:333–60.

[40] Glenn N Cohort analysts futile quest-statistical attempts to separate age, period and cohort effects. Am Soc Rev 1976;41:900–4.

[41] Woodward A, Kawachi IJ Why reduce health inequalities? J Epidemiol Community Health 2000;54:923–9. 11076989 10.1136/jech.54.12.923 PMC1731601

