# Supplementary material for: Cohort differences in the levels and trajectories of frailty among older people in England
Source: J Epidemiol Community Health. 2015 Feb 2;69(4):316–21. doi: 10.1136/jech-2014-204655 (PMC4392235; doi:10.1136/jech-2014-204655)
Supplement: Web supplement [file jech-2014-204655-s1.pdf]

Supplementary material

**Description**	**Assigned values** (value of 1 indicates a deficit and 0 no deficit)					
Difficulty with walking 100 yards	No=0	Yes=1				
Difficulty sitting for about two hours	No=0	Yes=1				
Difficulty getting up from a chair after sitting for long periods	No=0	Yes=1				
Difficulty climbing several flights of stairs without resting	No=0	Yes=1				
Difficulty climbing one flight of stairs without resting	No=0	Yes=1				
Difficulty stooping, kneeling, or crouching	No=0	Yes=1				
Difficulty reaching or extending arms above shoulder level	No=0	Yes=1				
Difficulty pulling or pushing large objects like a living room chair	No=0	Yes=1				
Difficulty lifting or carrying weights over 10 pounds, like a heavy bag	No=0	Yes=1				
Difficulty picking up a 5p coin from a table	No=0	Yes=1				
Difficulty dressing, including putting on shoes and socks	No=0	Yes=1				
Difficulty walking across a room	No=0	Yes=1				
Difficulty bathing or showering	No=0	Yes=1				
Difficulty eating, such as cutting up your food	No=0	Yes=1				
Difficulty getting in or out of bed	No=0	Yes=1				
Difficulty using the toilet, including getting up or down	No=0	Yes=1				
Difficulty using a map to figure out how to get around in a strange place	No=0	Yes=1				
Difficulty preparing a hot meal	No=0	Yes=1				
Difficulty shopping for groceries	No=0	Yes=1				
Difficulty making telephone calls	No=0	Yes=1				
Difficulty taking medications	No=0	Yes=1				
Difficulty managing money, (e.g paying bills and keeping track of expenses)	No=0	Yes=1				

Description	**Assigned values** (value of 1 indicates a deficit and 0 no deficit)					
Difficulty doing work around the house or garden	No=0	Yes=1				
Self-reported general health	Excellent=0	V.good=0.25	Good=0.5	Fair=0.75	Poor=1	
Whether respondent has felt depressed much of the time during past week	No=0	Yes=1				
Whether respondent felt everything they did during the past week was an effort	No=0	Yes=1				
Whether respondent felt their sleep was restless during the past week	No=0	Yes=1				
Whether respondent was happy much of the time during the past week	Yes=0	No=1				
Whether respondent felt lonely much of the time during the past week	No=0	Yes=1				
Whether respondent enjoyed life much of the time during the past week	Yes=0	No=1				
Whether respondent felt sad much of the time during the past week	No=0	Yes=1				
Whether respondent could not get going much of the time during the past week	No=0	Yes=1				
High blood pressure or hypertension (self-reported)	No=0	Yes=1				
Angina (self-reported)	No=0	Yes=1				
Heart attack (including myocardial infarction or coronary thrombosis) (self-reported)	No=0	Yes=1				
Congestive heart failure (self-reported)	No=0	Yes=1				
An abnormal heart rhythm (self-reported)	No=0	Yes=1				
Diabetes or high blood sugar (self-reported)	No=0	Yes=1				
A stroke (cerebral vascular disease) (self-reported)	No=0	Yes=1				
Chronic lung disease such as chronic bronchitis or emphysema (self-reported)	No=0	Yes=1				
Asthma(self-reported)	No=0	Yes=1				
Arthritis (including osteoarthritis , or rheumatism) (self-reported)	No=0	Yes=1				

**Description**	**Assigned values** (value of 1 indicates a deficit and 0 no deficit)					
Osteoporosis, sometimes called thin or brittle bones (self-reported)	No=0	Yes=1				
Cancer or a malignant tumour (excluding minor skin cancers) (self-reported)	No=0	Yes=1				
Parkinson's disease (self-reported)	No=0	Yes=1				
Any emotional, nervous or psychiatric problems (self-reported)	No=0	Yes=1				
Alzheimer's disease (self-reported)	No=0	Yes=1				
Dementia, organic brain syndrome, senility or any other serious memory impairment (self-reported)	No=0	Yes=1				
Self-reported eyesight (while using lenses, if appropriate)	Excellent=0	V.good=0.2	Good=0.4	Fair=0.6	Poor=0.8	Blind=1
Self-reported hearing (while using hearing aid if appropriate)	Excellent=0	V.good=0.25	Good=0.5	Fair=0.75	Poor=1	
Whether respondent has fallen down at all /last year /last 2years	No=0	Yes=1				
Whether respondent has fractured hip ever /in last 2 years	No=0	Yes=1				
Whether respondent has had joint replacement **ever**	No=0	Yes=1				
Whether respondent has had pain whilst walking	No=0	Yes=1				
Identify today's date: day of month	Yes=0	No=1				
Identify today's date: month	Yes=0	No=1				
Identify today's date: year	Yes=0	No=1				
Identify the day of the week?	Yes=0	No=1				
Passed prospective memory test	Yes=0	No=1				
Immediate word recall (sample organised into quartiles)	1st quartile=0	2nd quartile=0.3[illegible]	3rd quartile=0.6[illegible]	4th quartile=1		
Fluency test (animals) (sample organised into quartiles)	1st quantile=0	2nd quantile=0.25	3rs quantile=0.5	4th quantile=0.75	5th quartile=1	5th quartile=1
Delayed word recall (sample organised into quartiles)	1st quantile=0	2nd quantile=0.25	3rs quantile=0.5	4th quantile=0.75	5th quartile=1	5th quartile=1

Table S1: Deficits included in the English Longitudinal Study of Ageing frailty index and their coding

	Model 1 (cohort trajectories)	Model 2 (gender)	Model 3 (wealth)
Fixed effects			
**Intercept**	0·18***	0·17***	0·23***
**Cohort**	0·03***	0·03***	0·03***
**Cohort^2**	0·003***	0·003***	0·003***
**Growth rate**	0·01***	0·01***	0·01***
**Growth rate^2**	0·001***	0·001***	0·001***
**Growth rate*cohort**	0·005***	0·005***	0·005***
**Female (ref category; male)**		0·02*	
**Female*cohort**		0·004**	
**Middle wealth tertile**			-0·05***
**Richest tertile**			-0·08***
**Middle wealth tertile*cohort**			0·004**
**Richest tertile*cohort**			0·004**
**Middle wealth tertile*growth rate**			-0·0007
**Richest tertile*growth rate**			-0·002**
Random effects			
**Level 1: within person**	0·002***	0·002***	0·002***
**Level 2: In intercept**	0·01***	0·01***	0·01***
**Level 2: In growth rate**	0·0005***	0·0003***	0·0005***
Model fit			
**Log likelihood**	48,163	48,223	47,834
**AIC (smaller the better)**	-96,306	-96,424	-95,635
**BIC (smaller the better)**	-96,220	-96,320	-95,498
**Observations**	40,391	40,391	39,681

Table S2: Growth curve models of frailty and ageing by cohort, gender and wealth (quintiles)

*p<0.05; **p<0.01; ***p<0.001 (two-tailed tests)

AIC=Akaike information criterion. BIC=Bayesian information criterion. Standard error of the fixed-effect coefficients are not reported here. Random effects relate to the variance component. Whilst covariance components are estimated we do not report them here.

	Model 1 (cohort trajectories)	Model 2 (gender)	Model 3 (wealth)
Fixed effects			
Intercept	0·16***	0·14***	0·21***
Cohort	0·02***	0·02***	0·02***
Cohort^2	0·002***	0·001**	0·002***
Growth rate	0·01***	0·01***	0·01***
Growth rate^2	0·001***	0·001***	0·001***
Growth rate*cohort	0·004***	0·003***	0·003***
Female (ref category; male)		0·03***	
Female*cohort		0·005***	
Middle wealth tertile			-0·04***
Richest tertile			-0·08***
Middle wealth tertile*cohort			0·004*
Richest tertile*cohort			0·003
Middle wealth tertile*growth rate			-0·0002
Richest tertile*growth rate			-0·001
Random effects			
Level 1: within person	0·002***	0·002***	0·002***
Level 2: In intercept	0·009***	0·008***	0·007***
Level 2: In growth rate	0·0002***	0·0002***	0·0002***
Model fit			
Log likelihood	35,676	35,745	35,277
AIC (smaller the better)	-71,332	-71,467	-70,521
BIC (smaller the better)	-71,250	-71,369	-70,390
Observations	26,200	26,200	25,730

Table S3: Growth curve models of frailty and ageing by cohort, gender and wealth (quintiles) for sample members who have frailty index measures at all five waves of ELSA.

*p<0.05; **p<0.01; ***p<0.001 (two-tailed tests)

AIC=Akaike information criterion. BIC=Bayesian information criterion. Standard error of the fixed-effect coefficients are not reported here. Random effects relate to the variance component. Whilst covariance components are estimated we do not report them here.

Frailty trajectories: all people (aged 50+)

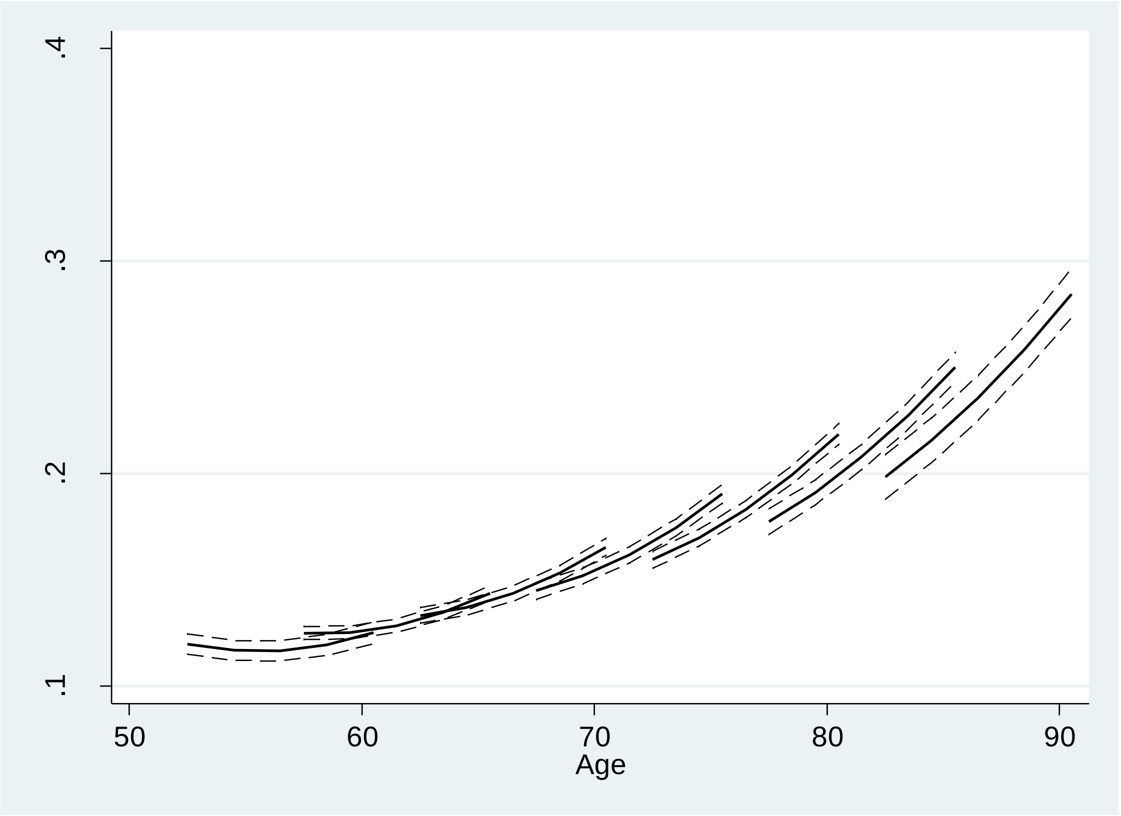

Frailty trajectories: by gender

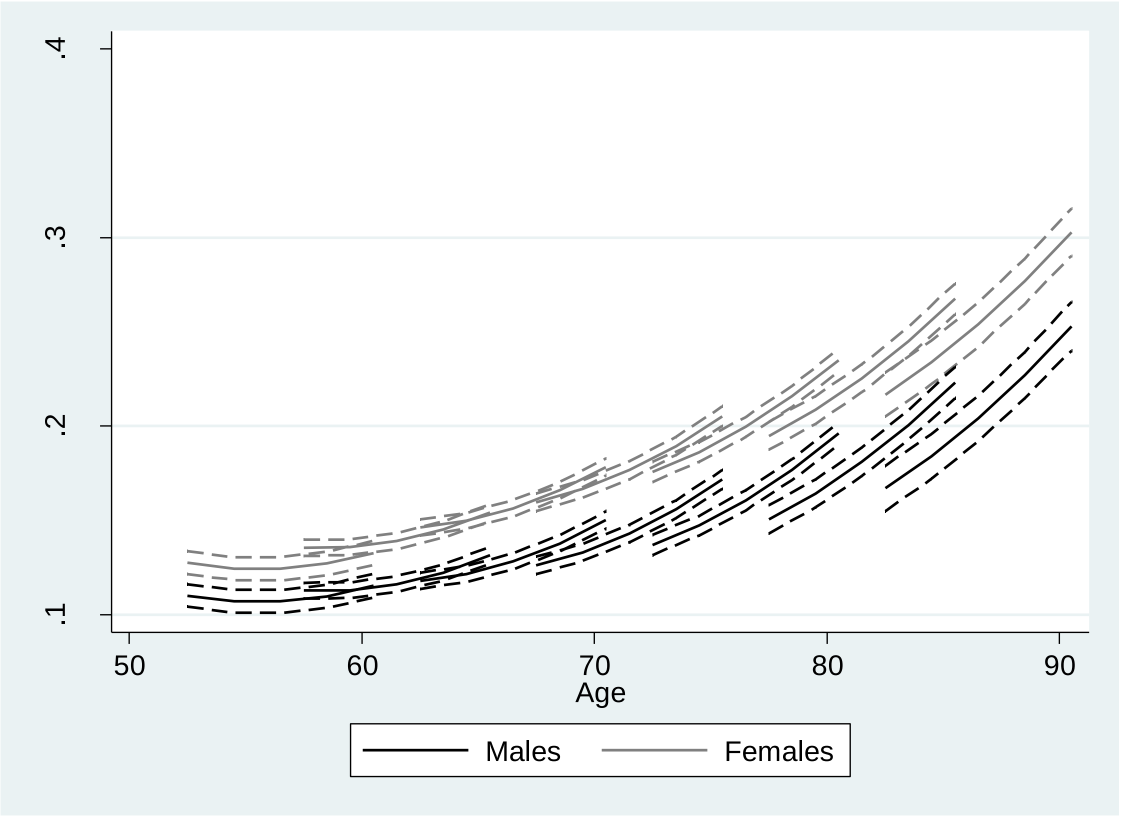

Continued overleaf

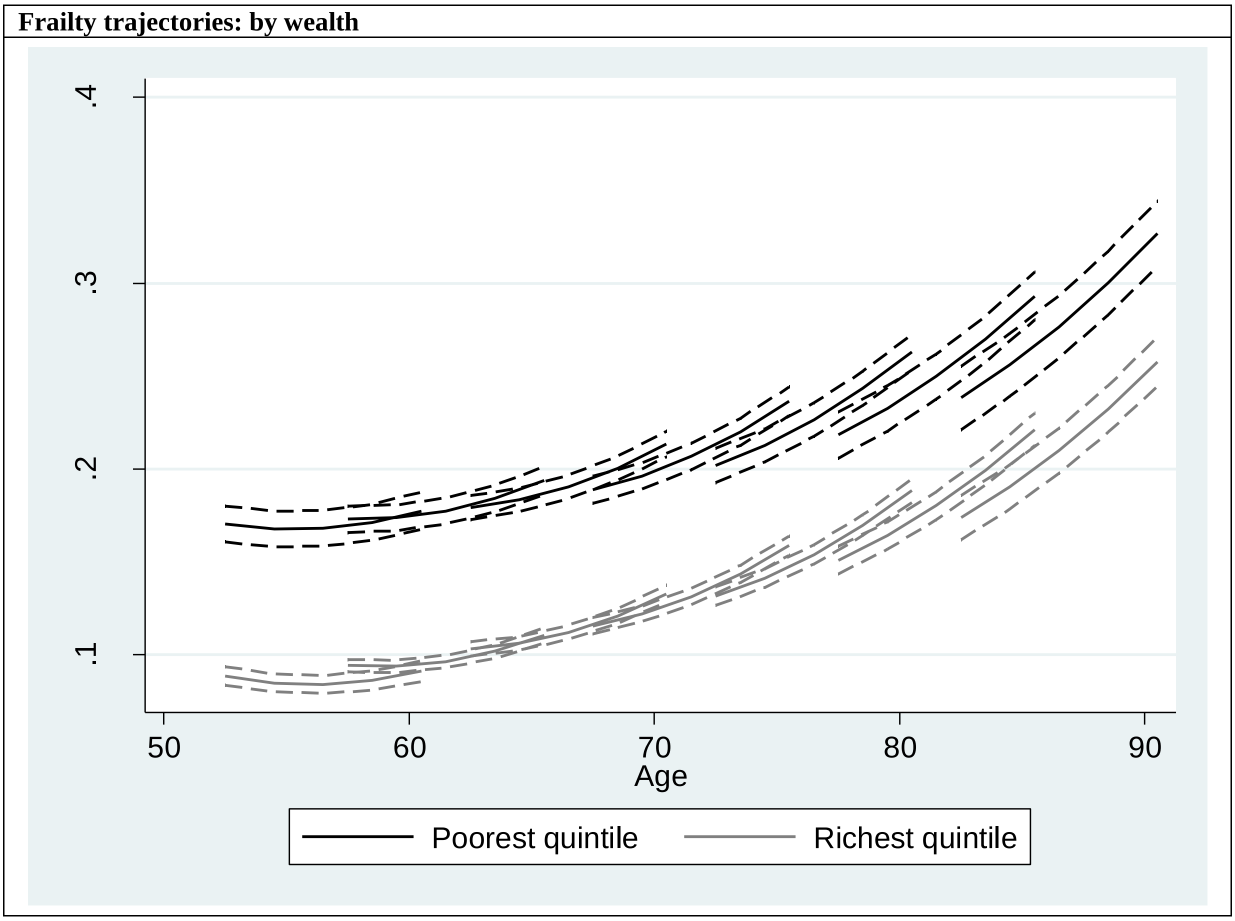

Figure S1: Model cohort specific trajectories of frailty by gender and wealth for the sample members who have frailty index measures at all five waves of ELSA.

Dotted lines indicate 95% confidence intervals around model estimates of frailty.

Missingness of frailty index by wave*	Frequency	%
_____	5,292	46·80
_2345	1,907	16·87
__345	1,194	10·56
___45	944	8·35
____5	698	6·17
___4_	227	2·01
__3__	169	1·49
_2___	141	1·25
__34_	120	1·06
_234_	112	0·99
_2_45	105	0·93
_23__	104	0·92
__3_5	69	0·61
_23_5	60	0·53
_2__5	46	0·41
_2_4_	32	0·28
1_345	25	0·22
1____	22	0·19
12_45	8	0·07
123__	6	0·05
1__45	6	0·05
1___5	5	0·04
1_3__	4	0·04
12___	3	0·03
12__5	2	0·02
1_34_	2	0·02
1__4_	2	0·02
1234_	1	0·01
123_5	1	0·01
	11,307	100

Table S4: Patterns of missingness in the frailty index (waves 1 to 5)

*_ indicates a frailty index is available for a particular wave whilst a numerical value indicates the wave for which the frailty index is missing.

Number of waves for which frailty index is missing	Number	%	Cumulative percentage
**0**	5,292	47	46
**1**	1,257	11	58
**2**	1,329	12	69
**3**	1,487	13	82
**4**	1,942	17	100
**Total**	11,307	100	100

Table S5: Number of waves of which the frailty index is missing (waves 1 to 5)

Status at wave 5	Frequency	%	Cumulative %
**Full or partial interview by proxy**	6,242	54·8	54·8
**Died**	1,047	9·2	64·0
**Ill**	215	1·9	65·9
**In Institution**	42	0·4	66·3
**Not issued**	1,188	10·4	76·7
**Out of Britain**	142	1·3	77·9
**Missing for other reason**	2,515	22·1	100·0

Table S6: Status at wave 5 including reasons for non-response

Coefficient	Odds Ratio	Std. Err.	z	P>z	95% confidence interval	
**Age (at wave 1 - 5 year intervals)**	1.01	0.00	6.37	<0.0000	1.01	1.02
**Female**	0.84	0.03	-4.22	<0.0000	0.78	0.91
**2nd poorest quintile of wealth**	0.86	0.05	-2.38	0.017	0.76	0.97
**Middle quintile of wealth**	0.73	0.05	-4.77	<0.0000	0.65	0.83
**2nd richest quintile of wealth**	0.64	0.04	-6.76	<0.0000	0.56	0.73
**Richest wealth quintile**	0.54	0.04	-9.13	<0.0000	0.47	0.61
**Frailty index**	2.01	0.39	3.55	<0.0000	1.37	2.95
**Constant**	0.26	0.04	-8.81	<0.0000	0.20	0.36

Table S7: Coefficients from a logistic regression model predicting non-response (at any wave between wave 2 and wave 5)
